# Temporal dynamics of heart rate variability reveal stressor-specific autonomic patterns: a multi-stressor study

**DOI:** 10.3389/fnins.2026.1832059

**Published:** 2026-07-29

**Authors:** Bérangère Villatte, Danielle Benesch, Alain Vinet, Rachel E. Bouserhal, Jérémie Voix, Sylvie Hébert

**Affiliations:** 1 École d’orthophonie et d’audiologie, Faculté de médecine, Université de Montréal, Montréal, QC, Canada; 2 Centre for Research on Brain, Language and Music-CRBLM, Montréal, QC, Canada; 3 Centre de recherche, Hôpital du Sacré-Coeur-de-Montréal, Montreal, QC, Canada; 4 École de Technologie Supérieure, Université du Québec, Montréal, QC, Canada; 5 Département de pharmacologie et physiologie, Faculté de médecine, Université de Montréal, Montréal, QC, Canada

**Keywords:** autonomic nervous system, chronic stress, heart rate variability, laboratory stress, noise sensitivity, noise stress

## Abstract

**Introduction:**

Laboratory stress paradigms provide controlled conditions to study autonomic stress responses and their relevance to health and disease. The autonomic nervous system adapts dynamically during stress, yet traditional heart rate variability (HRV) averages overlook rapid allodynamic adjustments. Few studies have characterized within-task HRV dynamics across multiple stressors or considered individual stress-related traits. This study examined fine-grained HRV responses to mental, noise, and pain stressors and assessed how perceived chronic stress and noise sensitivity contribute to these autonomic patterns.

**Method:**

Thirty healthy adults completed mental arithmetic, noise exposure, and cold pressor tasks while electrocardiogram (ECG) was recorded. HRV (MeanNN, RMSSD) was computed in 30-s windows, and perceived stress and noise sensitivity were assessed with validated questionnaires.

**Results:**

Mental stress elicited the largest global HRV reductions, pain affected only MeanNN, and noise produced no global changes. However, time-resolved analyses revealed distinct HRV dynamics across all stressors that were not captured by global averages. Chronic stress and noise sensitivity had little effects on global HRV but significantly moderated fine-grained responses to mental and noise stress during anticipation, stress, or recovery phases.

**Discussion:**

Autonomic stress reactivity is best understood through its temporal dynamics, as global HRV metrics obscure stressor-specific responses and the influence of individual vulnerability. Temporal analyses revealed distinct autonomic signatures for mental, noise, and pain stressors, indicating that they may engage partially distinct autonomic pathways and should not be treated as interchangeable experimental proxies of “stress.” The modulation by chronic stress and noise sensitivity highlights the importance of integrating trait-level factors when modeling autonomic adaptation. In line with the allodynamic framework, these findings suggest that high-resolution temporal analyses may provide a relevant approach for studying autonomic stress responses and their links to environmental stress exposure and health.

## 1 Introduction

Laboratory stress challenges are essential tools for investigating physiological responses to stress in both health and disease. They enable standardized stress exposure, allowing precise control over intensity, duration, and context, and thereby help isolate causal mechanisms by reducing confounding factors present in naturalistic environments. Moreover, they provide mechanistic insights into stress regulation that is critical for identifying vulnerability to stress-related disorders and for guiding targeted interventions. In this respect, stress challenge paradigms have been valuable for predicting cardiovascular risk (Lovallo, 2010), differentiating psychiatric profiles (Giles et al., 2014), and revealing metabolic and inflammatory dysregulation associated with depression and chronic stress (Jurgens et al., 2023).

The autonomic nervous system (ANS) activation unfolding over seconds to hours constitutes an adaptive response to environmental demands. Under normal conditions autonomic activity returns toward baseline once the stressor has ended. However, repeated or chronic exposure to stress may alter autonomic regulation and contribute to cardiovascular and other health consequences (McEwen, 2004).

Sympathetic activation rapidly upregulates cardiac, vascular, sudomotor, and other effectors within the fight-or-flight response, whereas parasympathetic activity, typically manifested through rapid heart rate variations and respiratory sinus arrhythmia, concomitantly declines. Autonomic activity can be indexed through heart rate variability (HRV), which quantifies beat-to-beat fluctuations in cardiac activity. Although HRV reactivity is commonly assessed using values averaged across an entire stress task, such global measures may obscure the dynamic and adaptive nature of autonomic regulation (Berntson et al., 2016; Kim et al., 2018) Contemporary models emphasize that sympathetic and parasympathetic branches interact in complex and sometimes non-linear ways, particularly during acute stress (Thayer and Lane, 2000; Billman, 2013).

Within neurovisceral integration frameworks, autonomic regulation comprises both a tonic regulatory capacity and a dynamic component governing moment-to-moment adjustments to environmental demands (Thayer and Lane, 2009). Consequently, while global HRV indices remain useful for summarizing overall autonomic modulation during stress (Task Force of the European Society of Cardiology the North American Society of Pacing Electrophysiology, 1996; O’ Riordan et al., 2023), they should not be interpreted as standalone predictors of clinical cardiovascular outcomes. Rather, in the present context, HRV is used as a non-invasive marker or autonomic regulation, with time-resolved analyses providing additional insight into the temporal organization, flexibility, and efficiency of stress adaptation. This perspective is consistent with evidence highlighting the complex and non-linear nature of autonomic regulation (Goldberger et al., 2002; Shaffer and Ginsberg, 2017).

Because stress adaptation unfolds over time, examining temporal HRV trajectories may offer a sensitive means of detecting alterations associated with allostatic load. However, relatively few studies have investigated within-task HRV dynamics, and those that have typically relied on a single stress paradigm (Nagy et al., 2015; Pereira et al., 2017; Rosenblum et al., 2025). These studies demonstrate that time-resolved analyses can reveal phase-specific autonomic responses that are not apparent in traditional pre- versus post-stress comparisons (Nagy et al., 2015). Moreover, evidence suggests that different stressors may evoke distinct temporal patterns of activation and recovery (Nagy et al., 2015). Nevertheless, previous work has generally examined different stressors in separate participant samples, leaving unresolved how autonomic regulation unfolds across different stress modalities within the same individuals.

To address this gap, the present study examines the HRV dynamics across three distinct stressors – mental, noise, pain – in a within-subject design. Consistent with previous work, we included a mental calculation task (cognitive stressor), which requires high attentional engagement and is known to elicit robust sympathetic activation and vagal withdrawal, and a cold pain task (physical stressor), which reliably induces strong autonomic arousal through nociception and vasoconstriction. Based on previous HRV findings (Crosswell and Lockwood, 2020), cortisol studies (McRae et al., 2006), and the results reported by Nagy et al. (2015), we hypothesized that mental stress would evoke a stronger autonomic response than pain.

We also incorporated a noise-stress task (psychophysical stressor) to assess reactivity to an ecologically relevant sensory stressor characterized by perceptual load, unpredictability, and aversiveness, features that may engage autonomic processes distinct from those elicited by cognitive or nociceptive stress. Chronic exposure to moderate-intensity noise has been associated with poorer self-reported health (Kodji et al., 2023), adverse clinical outcomes such as diabetes, depression, and insomnia (Park et al., 2017), and increased cardiovascular and mental health risk (Minkin et al., 2025) likely mediated in part by autonomic dysregulation. Establishing a controlled laboratory model of noise exposure is therefore essential for understanding how autonomic responses are shaped by both acoustic and individual differences in noise sensitivity. Given the limited number of studies examining ANS responses to noise under controlled conditions, our hypothesis regarding noise-induced autonomic activation was exploratory and probabilistic rather than strongly theory-driven (Park et al., 2018; Crosswell and Lockwood, 2020; Roddick et al., 2025).

To capture rapid autonomic adjustments during acute stressors, we adopted a time-resolved HRV approach based on 30-s windows. This strategy requires indices that remain valid under ultra-short and non-stationary conditions. Although frequency-domain and non-linear measures may provide complementary information, their validity in such conditions remains less established (Task Force of the European Society of Cardiology the North American Society of Pacing Electrophysiology, 1996; Shaffer and Ginsberg, 2017). Frequency-domain analyses, in particular, require dedicated time-frequency methods and are therefore addressed separately in a companion study (Villatte et al., 2026). Accordingly, the present study focused on the time-domain metrics RMSSD and MeanNN, both of which have demonstrated robust estimation and physiological interpretability in windows as short as 10–30 s (McNames and Aboy, 2006; Salahuddin et al., 2007; Nussinovitch et al., 2011; Baek et al., 2015).

By tracking RMSSD and MeanNN across successive task phases, we aimed to characterize the magnitude, timing, and recovery of autonomic responses, key features of adaptive stress regulation and resilience (Phillips et al., 2013; Roddick et al., 2025). Within allostatic frameworks, acute stressors normally elicit flexible autonomic adjustments that support coping and behavioral adaptation, whereas chronic stress exposure may progressively alter regulatory capacity and stress responsiveness (McEwen, 1998). In light of this framework, we also explored whether perceived chronic stress and noise sensitivity moderated ANS responses. We hypothesized that individuals reporting higher levels of perceived chronic stress would exhibit altered autonomic response dynamics, including reduced, delayed, or less flexible reactions to acute stressors. In contrast, because noise sensitivity has been associated with heightened autonomic responses to aversive sounds (Walker et al., 2016; Park et al., 2018), we expected higher noise sensitivity to predict greater reactivity during the noise-stress task.

## 2 Materials and methods

### 2.1 Participants

Thirty healthy young adults (15F, 15M) aged of 27.2 ± 3.9 years were recruited. Inclusion criteria included normal hearing thresholds (≤15 dB HL at 0.5–4 kHz), normal loudness discomfort levels (≥90 dB HL at 0.5–8 kHz), and good self-reported psychological and physical health. All participants had a normal body mass index (22.8 ± 3.0). Exclusion criteria were diagnosed depression or anxiety disorders, medication affecting stress or cardiac function, hearing-related disorders, and any conditions affecting hearing on the day of the experiment (e.g., cold, sinusitis, Covid-19). Participants with Raynaud’s disease, cerebral, respiratory, cardiovascular diseases, diabetes, hypoglycemia, or pregnancy were also excluded. The study was approved by the University’s Institutional Review Board. All participants provided written informed consent.

### 2.2 Stress experiment

#### 2.2.1 Mental task

**FIGURE 1:**
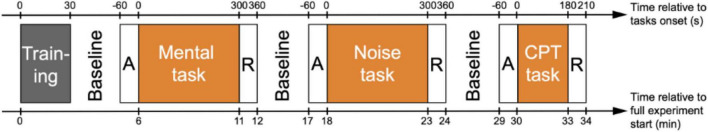
Stress induction procedure. A, anticipation; CPT, Cold Pressor Test; R, recovery.

A silent version of the arithmetic task from the Trier Social Stress Test (Kirschbaum et al., 1993) was developed using MATLAB R2020b and PsychToolbox (v3.0.16). Participants were instructed to count backward from 1022 in steps of 13 as quickly and accurately as possible. The absence of social evaluation was compensated by additional stressful features such as response feedback (good, wrong, time-out) and a timer of 7.5 s for each answer. Errors or timeouts required restarting from 1022. Responses were entered on a mini number keyboard, and participants were asked to remain silent to minimize the influence of speech on breathing and heart rate. The task lasted 5 min and was preceded by a 30-s practice trial presented at the beginning of the experimental session, before the first rest period. The practice trial consisted of subtracting 13 from 1158 and was followed by a 5-min recovery period before the real task began (Figure 1).

#### 2.2.2 Noise task

A low-frequency noise of increasing intensity was generated using Audacity software (Muse Group, Limassol, Cyprus) and presented through two 45°-azimuth free-field loudspeakers using an AC40 audiometer. This noise has previously been associated with increased cortisol levels in healthy adults (Waye et al., 2002; Hébert and Lupien, 2009), and the increasing intensity with increased activity in the amygdala (Bach et al., 2008). Therefore, an exponential ramp of increasing intensity was applied at the beginning of the noise, increasing from 40 to 90 dBA for 2 min and remaining stable at 90 dBA for 3 min, for a total exposure of 5 min.

#### 2.2.3 Pain task

The Cold Pressor Test (CPT) is a pain task commonly used to induce physical stress (Mourot et al., 2009). It involves immersing the hand in cold water for as long as possible, typically with a maximum of 1 min. In the present study, the water temperature was set at 6.5 °C based on Mitchell (Mitchell et al., 2004) and our pilot data, which was the best compromise for most adults to be able to keep their hand immersed for the full task duration (3 min). Temperature was maintained using a 0.1 °C thermometer and a stirring pump to ensure continuous water circulation.

### 2.3 Physiological assessment

#### 2.3.1 ECG recording

A continuous electrocardiogram (ECG) was recorded with a clinical Holter monitor (Burdick Spacelabs, Deerfield, USA) using a 5-lead configuration. The sampling rate was set at 500 Hz. To synchronize the ECG with the onset and offset of each task, participants pressed a button on the Holter device, which inserted a marker on ECG trace. Signals were transferred to a computer for offline processing and band-pass filtered (0.01–100 Hz). For each heartbeat, R-peak was defined as the center of gravity of the QRS complex (Jacquemet et al., 2011). RR intervals were validated using Burdick Vision Premier Holter software (Cardiac Science, Waukesha, WI, USA). Artifacts were identified through a multi-step procedure: signals were bandpass filtered (0.01–100 Hz), QRS complexes detected automatically using a MATLAB algorithm (Dube et al., 1988), and all detections were manually reviewed by a trained physician to exclude noise segments, pauses, arrhythmias, and ectopic beats. Only normal beats were retained to compute normal-to-normal intervals, which were converted into inter-beat intervals (IBIs) for HRV analysis (MeanNN and RMSSD). Excluded data due to artifacts were minimal (0.95% of data for the noise task, 0.33% for the Pain task, and 0% for the Mental task). HRV metrics were computed for each time window using MATLAB (R2021a), where MeanNN corresponded to the arithmetic mean of all normal IBIs and RMSSD corresponded to the root mean square of successive differences between adjacent IBIs. Time-frequency analyses of the data are reported in a separate methodological paper (Villatte et al., 2026), which provides a complementary characterization of HRV dynamics using advanced analytical approaches.

#### 2.3.2 Task segmentation

The stress periods were extended to 60 s before and after the stress tasks to include anticipation and recovery periods. In total, the Mental and Noise tasks lasted 7 min (420 s), and the Pain task lasted 5 min (300 s). HRV metrics were calculated for 1) the entire stress interval (average stress response) as commonly reported in studies, and 2) over time windows of 30 s (time-varying stress response).

#### 2.3.3 Feature extraction

All features were extracted in the time domain. MeanNN was calculated as the mean duration of the NN intervals, and RMSSD was calculated as the root mean square of successive differences in the NN intervals. The values of these features were reported as the change (difference) from the individual baseline, which was the average of the 3 min taken in the middle of the rest period before each task.

#### 2.3.4 Psychometric assessment: chronic stress and noise sensitivity

Chronic stress was assessed using the French version of the 14-item Perceived Stress Scale (PSS-14; Lesage et al., 2012). The scales measure the degree to which life situations are appraised as stressful over the past month. It consists in 14 items rated on 5-points Likert-type scale ranging from 0 (never) to 4 (very often). Total score ranges from 0 to 56, where higher values represent greater perceived stress. Noise sensitivity was quantified using the validated Hyperacusis Questionnaire (HQ; Khalfa et al., 2002), a 14-item instrument designed to measure how noise affect daily situations according to attentional, social, and emotional dimensions. Total scores on the HQ range from 0 to 42, where higher values reflect greater levels of noise sensitivity. While clinical hyperacusis is often defined by cut-off scores of >22 (Aazh and Moore, 2017) or >28 (Khalfa et al., 2002), the scores in this study were treated as continuous variables and calculated as a mean averaged from the 14 individual items.

### 2.4 Procedure

Participants signed the informed consent form and were invited to sit in an audiometric booth. After a standard audiological evaluation, the stress experiment began. During Holter initialization (about 10 min), a gender-matched experimenter placed five electrodes on each participant. They were then briefly trained for the arithmetic task. Participants were then exposed to the three stress-inducing tasks in a predetermined order: Mental, Noise, and Pain, separated by 5 min of rest (Figure 1). This sequence ensured consistent stress conditions and minimized carry-over effects, particularly from the Pain task, which could cause pain to persist for several minutes. Verbal instructions were given at least 1 min before each task. Finally, participants completed four questionnaires and received financial compensation for their participation.

### 2.5 Statistical analyses

#### 2.5.1 Average stress response

To characterize global autonomic responses to stress, MeanNN and RMSSD values averaged over the baseline period and the entire stress period (including anticipation and recovery) were analysed using linear mixed-effects (LME) models (*lmer* R function). These models included Time (Baseline vs. Stress) and Task (Mental, Noise, Pain) as fixed effects, with Subject as random intercept. Least-squares means with Tukey correction were used for *post hoc* contrasts.

#### 2.5.2 Dynamic, fine-grained stress responses

To assess temporal variations in autonomic activity, HRV signals were decomposed into successive 30-s windows spanning anticipation, task execution, and recovery periods. LME models were used to test the main effect of Time, with Subject as random effect. Because task durations differed (Mental and Noise: 5 min; Pain: 3 min), resulting in different number of time windows, all fine-grained analyses were conducted separately for each task.

All LME models were controlled for sex.

#### 2.5.3 Effects of psychological traits

Associations between global change in RMSSD and MeanNN and psychological traits (PSS and HQ) were examined using Bayesian linear regressions (*BF*_10_ > 3 = moderate evidence; *BF*_10_ < 0.3 = evidence for null) and were conducted separately for each task.

To test whether psychological traits modulated HRV trajectories over time, task-specific LME models were used to examine Time × Trait interactions using 30-s time windows. These models were limited to three predictors in accordance with the One-in-Ten rule (Chowdhury and Turin, 2020), and moderation effects were estimated using the emtrends R function.

### 2.6 Assumptions check

Conditional model residuals were visually inspected for large deviations from normality using Q-Q plots and for heteroscedasticity using residual-versus-fitted-value plots, in accordance with recommendations for linear mixed-effects models (Pinheiro, 2000; Gelman and Hill, 2006; Schielzeth et al., 2020). Multicollinearity among fixed effects was assessed using variance inflation factors (VIFs), following standard guidelines for mixed-effects modeling (Zuur et al., 2010) Potential undue influence of observations on fixed-effect estimates was evaluated using Cook’s distance computed at the whole-model level, as implemented in model-based diagnostic tools for mixed-effects models (Lüdecke et al., 2021).

Across all fitted models, diagnostics did not indicate substantial deviations from residual normality or homoscedasticity, fixed effects were not affected by multicollinearity (all VIFs = 1), and no influential observations were detected. Accordingly, all observations were retained for analysis.

### 2.7 Carry-over effects

To assess potential order effects, baseline MeanNN and RMSSD values were compared across tasks using Bayesian analyses. Results indicated moderate evidence in favor of the absence of baseline differences across tasks (BF_10_ = 0.23 ± 0%), suggesting that recovery periods were sufficient to minimize carry-over effects.

#### 2.7.1 Power analysis

An *a priori* power analysis for within-subject effects (*n* = 30) indicated sensitivity to detect moderate-to-large effects (power = 0.78) at α = 0.05, supporting the analyses of main temporal effects^1^. Analyses examining the moderation effects of HQ and PSS on temporal trajectories were conducted on an exploratory basis.

## 3 Results

### 3.1 Perceived chronic stress and noise sensitivity

Considering the established HQ cut-off score of 22 for clinical hyperacusis, participants were relatively low sensitive to noise (mean HQ = 14.7 ± 5.90) (Table 1). The PSS-14 has no cut-off criteria, but the mean perceived stress (31.3 ± 5.8) can be considered moderate to high in our sample.

**TABLE 1:** Mixed-effects analyses showing main effect of time on MeanNN and RMSSD for each stress task (Satterthwaite-estimated degrees of freedom).

Task	Feature	NumDF	DenDF	F	*p*-value
Mental task	MeanNN	14	406	18.29	2.94e–35
	RMSSD	14	406	8.68	1.48e–16
Noise task	MeanNN	14	402.08	8.6	2.23e–16
	RMSSD	14	401.85	2.03	0.015
Pain task	MeanNN	10	286.92	4.08	2.93e–05
	RMSSD	10	287.1	1.91	0.0438

### 3.2 Baseline comparisons

Baseline MeanNN and RMSSD values were compared across tasks and task sequence. Bayesian analysis revealed *BF*_10_ = 0.23 ± 0% for both MeanNN and RMSSD, providing moderate evidence that baseline values did not systematically change across tasks. This indicates that recovery periods were sufficient to minimize carry-over effects due to fixed task order.

### 3.3 Average stress responses

**FIGURE 2:**
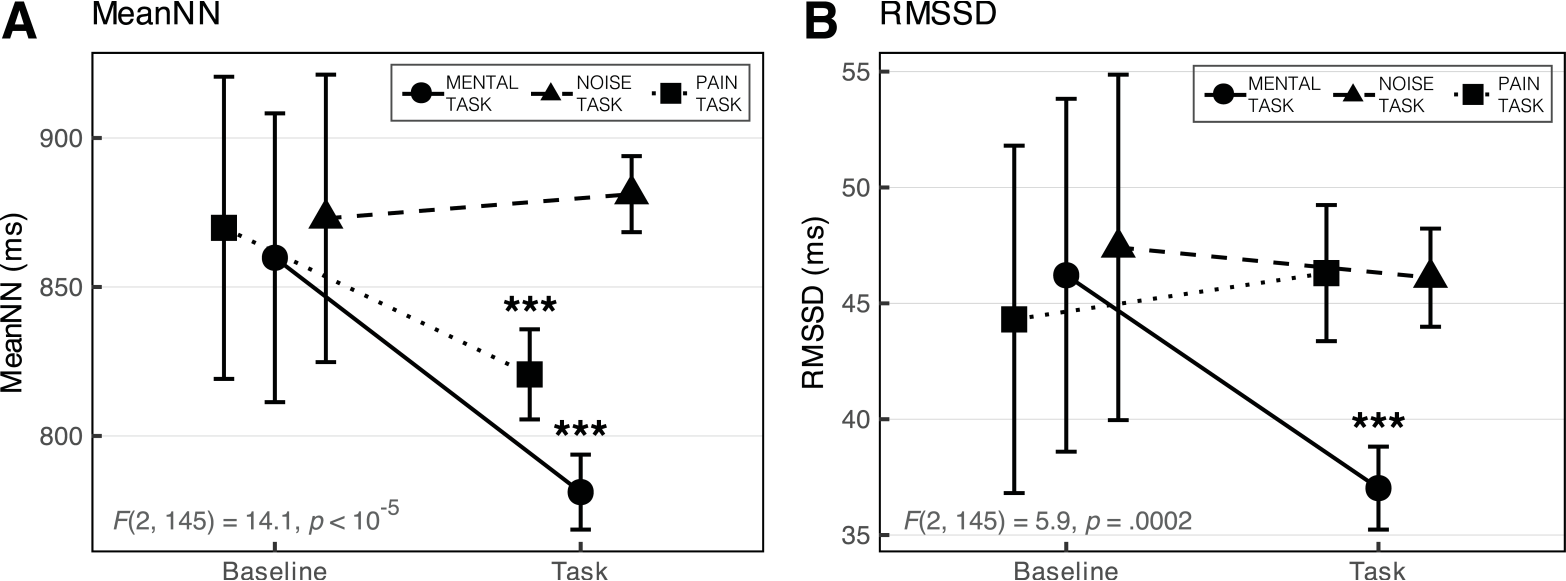
Non-normalized (absolute) **(A)** MeanNN and **(B)** RMSSD (Mean ± 95% CI) during the Mental, Noise and Pain tasks. The Mental stress induced the largest changes in both metrics. Pain led to reduction in MeanNN with no effect on RMSSD. Noise had no measurable effect on either feature. Black stars indicate significant pairwise comparison (Tukey-corrected; ***p < 0.001). MeanNN, mean of NN intervals; RMSSD, root mean square of successive differences in NN intervals.

Figure 2 shows, for each task, the non-normalized (absolute) MeanNN and RMSSD values averaged over baseline and over the entire stress period, including anticipation and recovery. MeanNN and RMSSD were analysed using linear mixed-effects models including Time (baseline, stress) and Task (mental, noise, pain).

There was a significant Time × Task interaction for both MeanNN (*F*(2, 145) = 14.1, *p* < 10^–5^) and RMSSD (*F*(2, 145) = 5.9, *p* = 0.004). Mental stress induced the largest changes, with significant decreases in both MeanNN and RMSSD relative to baseline (MeanNN: −78.7 ± 11.7 ms, *p* < 0.0001; and RMSSD: −9.2 ± 2.4 ms, *p* = 0.0002). Pain stress was associated with a decrease in MeanNN (LS-means = −48.80 ± 11.7 ms, *p* = 0.0001), whereas Noise stress but not RMSSD (*p* = 0.39). Noise stress did not significantly affect either feature (MeanNN, *p* = 0.50; RMSSD, *p* = 0.61).

### 3.4 Dynamic (fine-grained) response

**FIGURE 3:**
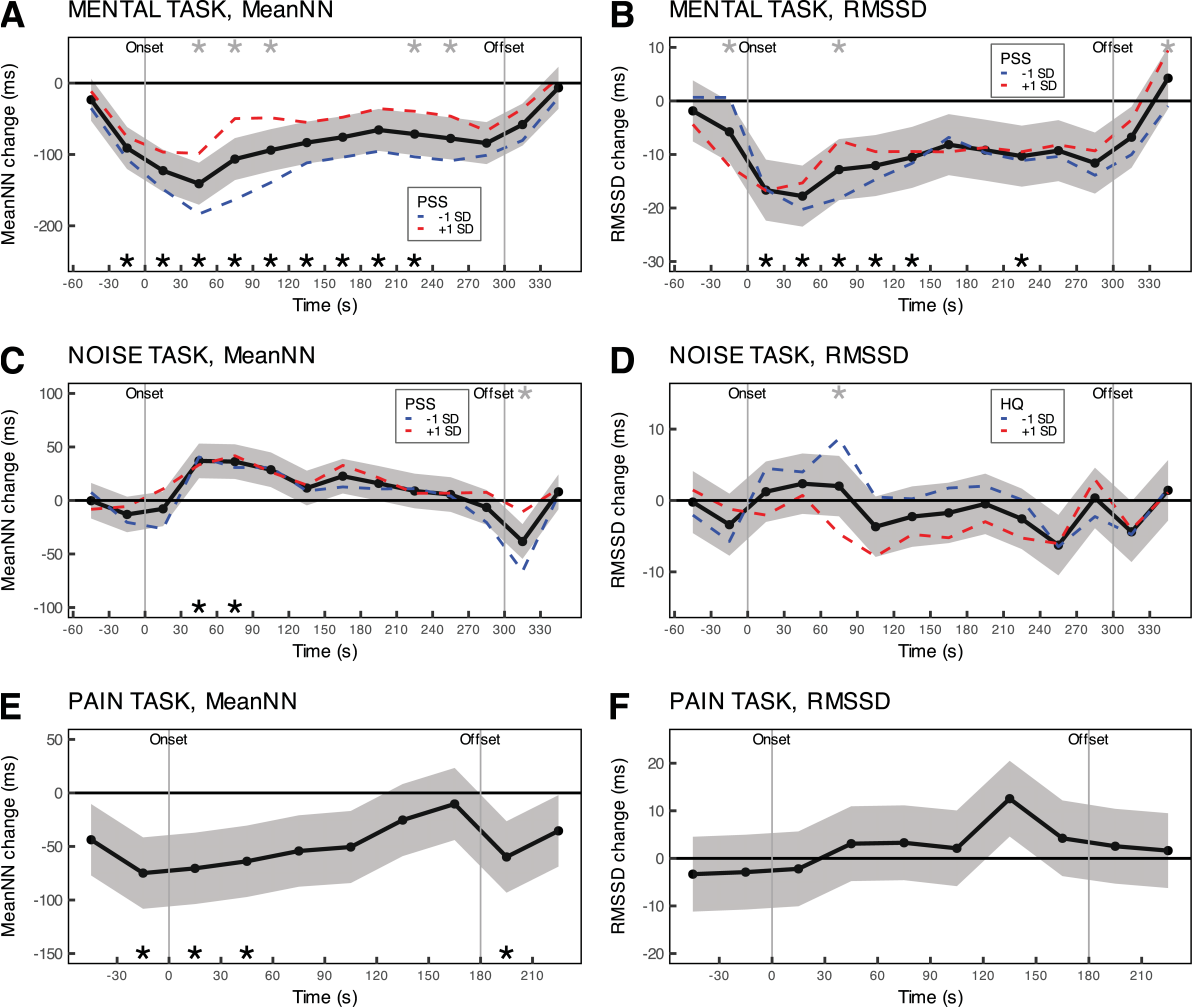
Time course of MeanNN and RMSSD across the three stress tasks. Autonomic responses are time-dependent and task-specific. Mental stress elicits the strongest changes **(A,B)**. Higher perceived chronic is associated with reduced autonomic reactivity during Mental stress **(A,B)** and noise recovery **(C)**, whereas higher noise sensitivity is associated with enhanced reactivity during noise exposure **(D)**. No trait-related effects are observed during the Pain task **(E,F)**. Black lines represent mean changes from baseline (±SE) for the whole sample, with black stars indicating significant deviations from baseline (Tukey-corrected). Colored lines show model-estimated trajectories at ± 1 SD of the moderator (PSS or HQ), illustrating its effect on temporal dynamics. *Gray stars indicate significant moderator at specific time points. MeanNN, mean of NN intervals; RMSSD, root mean square of successive differences in NN intervals.

Figure 3 (black lines) shows the temporal evolution of HRV features from anticipation through recovery using successive 30-s windows, aligned to task onset to highlight changes over time. Because task durations differed, dynamic analyses were conducted separately for each task. Mixed-effects models with Time as a within-subject factor revealed significant main effects of Time on both MeanNN and RMSSD across all stress tasks (Table 1), indicating non-stationary autonomic responses over time. Specifically, MeanNN exhibited robust temporal modulation during the Mental arithmetic, Noise, and Pain tasks (*F* range = 4.08–18.29). RMSSD also showed significant, though smaller, time-dependent effects across tasks (*F* range = 1.91–8.68).

More specifically, Mental stress was characterized by rapid and sustained autonomic changes, producing the largest effects on MeanNN and RMSSD at almost all time points (from −30 s to 300 s, 10^–3^ < *p*s < 10^–14^). As illustrated in Figures 3A,B, the largest effects occurred shortly after task onset, between −30 s and 90 s. In contrast, while no change was observed with the averaged analysis, inspection of the fine-grained time course revealed that Noise exposure was associated with an increase in MeanNN during the first 2 min following onset (0.009 < *p*s < 0.011; Figure 3C). RMSSD changes during Noise stress did not survive pairwise comparisons. During Pain stress, MeanNN decreased between −30 s and 60 s and again at 180 s after task onset (0.0005 < *p*s < 0.048), whereas RMSSD remained unchanged (Figures 3E, F).

Sex, included as a control variable in all models, was significant for RMSSD during Pain stress (*F*(1, 28) = 7.3, *p* = 0.023), with larger decreases in females relative to baseline compared to males.

### 3.5 Effects of chronic stress and noise sensitivity

Bayesian linear models were used to test associations between global MeanNN and RMSSD (averaged over time) and PSS or HQ scores, separately for each task. Except for moderate evidence that PSS was associated with MeanNN during the Mental task (BF_10_ = 4.2 ± 0%), evidence for other associations was null (0.35 < BF_10_ < 0.78).

Trait moderations were therefore examined on the temporal patterns, to assess whether individual PSS or HQ influenced stress responses when decomposed into 30-s windows. In Figure 3, this modulation is reflected by colored trajectories corresponding to + 1 SD (red lines) or -1SD (blue lines) of the moderator, with grey stars marking time points where the interaction is significant.

During Mental stress (Figures 3A,B), PSS moderated the temporal dynamics of both MeanNN (Time × PSS, *F*(14, 392) = 2.57, *p* = 0.002) and RMSSD (Time × PSS, *F*(14, 392) = 2.24, *p* = 0.006). Higher perceived chronic stress was associated with less pronounced reductions relative to baseline, particularly around task onset (30–90 s) and before task offset (210–240 s), as reflected by a clear separation of the trajectories during these intervals.

During Noise stress (Figures 3C, D), PSS moderated MeanNN (Time × PSS, *F*(14, 388) = 1.9, *p* = 0.025) during the recovery from stress, between 300 and 330 s. Individuals with lower perceived chronic stress showed an increase in MeanNN (blue line) that was absent in those with higher perceived chronic stress. RMSSD responses were moderated by HQ (Time × HQ, *F*(14, 388) = 2.1, *p* = 0.011), especially between 60 and 90 s, where individuals with higher noise sensitivity exhibited exaggerated RMSSD decreases compared with those with lower sensitivity, visible by an early separation of the trajectories.

No trait-related effects were observed during the Pain task.

## 4 Discussion

### 4.1 Temporal dynamics of autonomic stress reactivity

This study investigated the fine-grained temporal autonomic response evoked by mental, noise, and pain stressors. Our findings demonstrate that stress responses are dynamic rather than stationary and that averaging HRV across the entire stress task may obscure informative fluctuations in MeanNN and RMSSD. Notably, autonomic reactivity under noise exposure was detectable only in time-resolved analyses. Temporal HRV trajectories also revealed stressor-specific physiological patterns and were more sensitive to individual differences in perceived chronic stress and noise sensitivity than global HRV averages. Together, these findings highlight the value of high-resolution temporal analyses for characterizing autonomic stress reactivity across different stress modalities.

Across tasks, several common patterns emerged. For nearly all stressors and both HRV indices, we observed a rapid anticipatory shift within 30–60 s followed by a swift return toward baseline after stress offset. This anticipatory adjustment is consistent with allostatic models in which prefrontal-amygdalar circuits transiently withdraw parasympathetic influence to prepare for an upcoming challenge, whereas the rapid post-task rebound reflects efficient autonomic recovery (Roddick et al., 2025).

### 4.2 Stressor-specific autonomic patterns

In the Mental stress task, the marked global reductions in MeanNN and RMSSD confirmed a robust autonomic response consistent with previous TSST-like paradigms (Kim et al., 2018). However, analyses using 30-s windows revealed that the sustained decrease actually reached its peak within 30–90 s and returned to baseline within about 60 s after task offset. Our exploratory analyses suggested that perceived chronic stress may play a prominent role: individuals reporting higher stress exhibited hyporeactivity, showing smaller-than-expected reductions in RMSSD and attenuated MeanNN shifts. Blunted autonomic reactivity has been proposed as an early marker of allostatic load, reflecting a system less capable of mounting adaptive responses after prolonged strain (Vaccarino and Bremner, 2024)

Autonomic reactivity to Noise exposure was only detectable in dynamic HRV. Although amplitudes were small, MeanNN changes were observable during anticipation, the first 2 min of noise exposure, and recovery. Unlike Mental and Pain stress responses, MeanNN increased from baseline as noise intensity increased and returned to baseline once noise reached 90 dB SPL. These modest effects likely reflect limitations of the present noise protocol. Previous studies reporting endocrine responses to noise have generally relied on longer exposures (∼30 min), which were not feasible here (Bach et al., 2008; Hébert and Lupien, 2009; Fouladi et al., 2012). The development of laboratory paradigms capable of reliably eliciting autonomic responses to noise therefore remains an important challenge for future research (Babisch, 2002).

Unlike mental arithmetic and cold pain stress, noise exposure constituted a largely passive condition requiring limited attentional engagement. Such passive listening generally evokes weaker autonomic activation (Bosch et al., 2003) and may explain the relatively modest group-level effects observed here. However, this apparent passivity was not uniform across participants (Bosch et al., 2003). Although noise-sensitive individuals perform normally in quiet environments (Belojevic et al., 1992; Song et al., 2022), they show performance decrements at moderate noise levels of 55–75 dB. Consistent with this interpretation, individuals with higher noise sensitivity exhibited greater vagal withdrawal as noise intensity increased, as indexed by RMSSD decreases, whereas those with lower sensitivity showed minimal autonomic changes. This finding suggests that the noise stimulus was physiologically meaningful for at least a subset of participants and supports the view that noise sensitivity may reflect increased vulnerability to environmental stressors.

During the cold pain task, both global and temporal analyses detected changes in MeanNN, but no change was observed in RMSSD. It is possible that sex, which was significantly associated with overall RMSSD change when included as a covariate, contributed to opposing response tendencies across sexes that attenuated the effect at a group level. However, as the study was not specifically designed or powered to examine sex-dependent dynamics, this interpretation remains speculative and warrants investigation in larger samples. In contrast to noise and mental arithmetic stress, pain responses were not associated with either perceived chronic stress or noise sensitivity. This relative invariance suggests that cold pressor responses may be less sensitive to individual differences related to stress accumulation.

### 4.3 Neural mechanisms of stress-evoked autonomic responses

Few studies have examined the temporal evolution of autonomic biomarkers during stress tasks. Pereira et al. (2017), for example, analysed HRV during the Trier Social Stress Test (TSST) using 50-s windows and found that time-domain indices (AVNN, RMSSD, SDNN, and pNN20) were more reliable markers of stress than frequency-domain metrics. Nagy et al. (2015) examined HRV and salivary alpha-amylase dynamics during cognitive and pain stressors and observed significant autonomic changes only in the cognitive stress conditions. Similarly, Rosenblum et al. (2025) reported transient HRV changes during the Maastricht Acute Stress Test, with RMSSD decreases confined to the middle phase of the task. Our study extends these findings by demonstrating that MeanNN and RMSSD time-courses are also sensitive to individual differences such as chronic stress and noise sensitivity when appropriate stress modalities are used.

These temporal patterns are broadly consistent with the engagement of a common salience-autonomic network involving the anterior cingulate cortex, insula, and amygdala. At the same time, different stressors likely recruit partially distinct regulatory processes. Mental stress may rely more on cortico-limbic networks involved in cognitive effort and executive control (Thayer et al., 2012), noise stress on auditory-limbic circuits related to salience and threat detection (Zald and Pardo, 2002), and cold pressor stress on nociceptive-autonomic pathways supporting defensive responses (Hohenschurz-Schmidt et al., 2020). Although these interpretations remain speculative, they may help explain the distinct HRV trajectories observed across stress modalities.

### 4.4 Limitations

In the present protocol, all participants completed the stress tasks in a fixed sequence (mental arithmetic > noise > cold pressor). This order was implemented to minimize potential carryover from the Cold Pressor Test, as pain-related autonomic and central responses are known to persist beyond task offset and may last up to 15–30 min (Clewett et al., 2013). By contrast, converging evidence indicates that vagal activity following psychosocial or cognitive stress can substantially recover within short intervals (∼5 min) (Roddick et al., 2025), consistent with the recovery periods used here. Although baseline HRV measures did not differ across tasks, suggesting adequate restitution of resting autonomic state, more subtle or cumulative effects related to task order, including fatigue, habituation, or anticipatory adaptation, cannot be fully excluded. Future studies will benefit from larger samples and alternative designs, including partial counterbalancing or separate task sessions, to further isolate stressor-specific effects from order-related dynamics.

In addition, the study was primarily powered to detect moderate-to-large effects, with statistical power focused on within-task temporal effects. Because task durations differed (and thus the number of time windows), temporal analyses were conducted separately for each task. While visually distinct task-specific response profiles were observed, formal comparisons of task dynamics were beyond the scope of the present study and warrant investigation in larger samples.

A further limitation concerns the interpretation of stressor-specific responses. Laboratory stressors are often treated as standardized challenges; however, their physiological impact is shaped not only by the objective characteristics of the task but also by individual appraisal, prior experiences, and personal relevance. For example, mental arithmetic may evoke concerns related to competence or performance in some individuals, whereas noise or pain may carry different subjective meanings depending on past experiences and contextual factors. Consequently, part of the interindividual variability observed in autonomic responses may reflect differences in stressor appraisal, subjective meaning, and physiological stress reactivity. Such factors may contribute to the associations observed with perceived chronic stress and noise sensitivity in the present study. Future studies combining physiological measures with assessments of subjective appraisal, perceived threat, or task-related emotions may help disentangle these influences.

## 5 Conclusion

In conclusion, stress-evoked HRV responses differed markedly across time and stressor modality. Time-resolved analyses revealed physiological patterns and individual differences that were not apparent in conventional global HRV measures, particularly during noise exposure. These findings support the use of high-resolution temporal approaches to characterize autonomic stress reactivity and highlight the importance of considering both stressor modality and individual vulnerability when investigating physiological responses to stress. More broadly, they suggest that autonomic stress responses are best understood as dynamic processes whose temporal organization carries information beyond that captured by conventional summary measures.

## Data Availability

The datasets presented in this study can be found in the following online repository: Sylvie Hébert. Temporal Dynamics of Heart Rate Variability Reveal Stressorspecific Autonomic Patterns: A Multi-stressor Study. V1 ed: Borealis; 2026. https://doi.org/10.5683/SP3/ZWHKDI.

## Footnotes

1
http://psychstat.org/rmanova
